# Germanium Sub-Microspheres Synthesized by Picosecond Pulsed Laser Melting in Liquids: Educt Size Effects

**DOI:** 10.1038/srep40355

**Published:** 2017-01-13

**Authors:** Dongshi Zhang, Marcus Lau, Suwei Lu, Stephan Barcikowski, Bilal Gökce

**Affiliations:** 1 Technical Chemistry I and Center for Nanointegration Duisburg-Essen (CENIDE), University of Duisburg-Essen, Universitätsstraße 7, 45141, Essen, Germany

## Abstract

Pulsed laser melting in liquid (PLML) has emerged as a facile approach to synthesize submicron spheres (SMSs) for various applications. Typically lasers with long pulse durations in the nanosecond regime are used. However, recent findings show that during melting the energy absorbed by the particle will be dissipated promptly after laser-matter interaction following the temperature decrease within tens of nanoseconds and hence limiting the efficiency of longer pulse widths. Here, the feasibility to utilize a picosecond laser to synthesize Ge SMSs (200~1000 nm in diameter) is demonstrated by irradiating polydisperse Ge powders in water and isopropanol. Through analyzing the educt size dependent SMSs formation mechanism, we find that Ge powders (200~1000 nm) are directly transformed into SMSs during PLML via reshaping, while comparatively larger powders (1000~2000 nm) are split into daughter SMSs via liquid droplet bisection. Furthermore, the contribution of powders larger than 2000 nm and smaller than 200 nm to form SMSs is discussed. This work shows that compared to nanosecond lasers, picosecond lasers are also suitable to produce SMSs if the pulse duration is longer than the material electron-phonon coupling period to allow thermal relaxation.

Since the pioneering reports of Fojtik *et al*.^1^ and Neddersen *et al*.^2^ and significant contributions of Mafuné *et al*.^3^,^4^ on laser ablation in liquid (LAL), LAL has been demonstrated to be a scalable^5^,^6^ method for microparticle and nanoparticle synthesis that allows precise size control of the synthesized colloidal nanoparticles over a wide range from several micrometers^7^ to ~1 nanometer^8^. Due to applicability to most elements of the periodic table^9^, LAL is suited to produce a large variety of nanomaterials, including elemental particles^10^, oxides^11^, carbides^12^ as well as nanoalloy particles^13^,^14^,^15^, nanoparticle-polymer composites^16^,^17^,^18^, doped and undoped phosphor NPs^19^ and magnetic-chains^20^,^21^. More recently, the LAL-derivative techniques of laser fragmentation in liquid (LFL) and laser melting in liquid (LML) were developed. LFL was pioneered by Kamat^22^ and significantly advanced by the groups of Hashimoto^23^,^24^,^25^ and Meunier^26^ while LML was developed by Koshizaki and coworkers^27^,^28^,^29^. These advances in laser synthesis and processing of colloids have led to nanoparticles for multiple applications, such as catalysis^30^, solar cell^31^, photoelectronics^27^, cell imaging^32^ and theranostics^33^.

Recent advance in LML has shown its strength in the synthesis of a large variety of SMSs such as Si^34^, Au^35^,^36^,^37^, B_4_C^38^, Fe^29^, Cu^29^, Ag^39^, CuO^40^, ZnO^28^,^41^. Employment of superfluid helium which offers unique extreme conditions, including low temperatures, ultralow viscosity, high thermal conductivity, and good transparency in the visible region, can even allow the formation of CeO_2_ SMSs from anisotropic materials^42^. Some of these LML-generated SMSs have been used for various applications according to their intrinsic features. For example, Wang *et al*. synthesized TiO_2_ hollow SMSs via LML and applied them as scattering layer in quantum dot-sensitized solar cells and successfully promoted 10% current of solar-to-electric conversion efficiency^43^. The same group also generated ZnO SMSs and assembled them into a highly sensitive UV photodetector, showing that the ligand-free “clean” surface together with the single crystalline feature of the SMSs were helpful to enhance electronic carrier mobility^27^. Despite the success in producing a large variety of SMSs via nanosecond (ns) LML where laser heating dominates the whole process due to the efficient electron-phonon coupling^44^, the feasibility to achieve SMSs is still challenging for ultrafast lasers (e.g. femtosecond (fs) or picosecond (ps)) due to their “cold ablation” characteristic. Recently, Koshizaki *et al*. reported that during LML the energy absorbed by the particle will be dissipated rapidly after the laser matter interaction following the temperature decrease which takes place within several tens of nanoseconds^45^. By increasing the pulse duration lower temperatures and higher thresholds for spherical particle formation are obtained.

Herein, Ge SMSs with diameter of 200~1000 nm are synthesized by LML of Ge micropowders in water and organic liquid using picosecond laser pulses. A liquid jet setup is utilized to produce Ge SMSs (as shown in Fig. 1d). After different liquid jet passages upon laser irradiation, absorption spectra as well as the size and morphology evolution of the Ge particles are characterized by UV-Vis extinction spectroscopy, analytical disc centrifugation (ADC), SEM and TEM. From these results, a size dependent mechanism of Ge SMSs is proposed to give new insight on how powders with sizes ranging from nanoscale to microscale respond to ps laser irradiation. In addition, the melting behavior of Ge particles under our experimental conditions is verified theoretically by comparing the energy input for each particle with those required for melting and evaporation. Both the experimental finding and theoretical calculation support the conclusion that ps lasers are able to produce SMSs when the pulse duration is longer than the electron-phonon coupling period of the materials.

## Results

**Figure 1:**
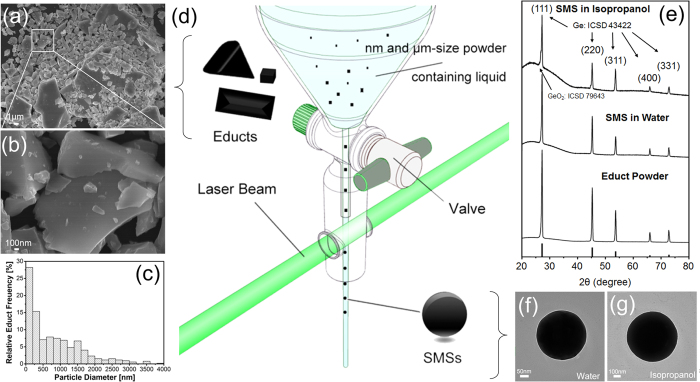
(**a,b**) SEM images of the educt Ge powders obtained by milling of Ge wafers (**c**) Primary particle size distribution of educt powders extracted from SEM images. (**d**) Experimental setup of LML using a liquid jet passage reactor. (**e**) XRD patterns of products obtained from laser irradiation of Ge powders in water and isopropanol as well as the XRD pattern of the educt powder. (**f,g**) TEM images of synthesized Ge SMSs after 100 liquid jet passages in water and isopropanol, respectively.

**Figure 2:**
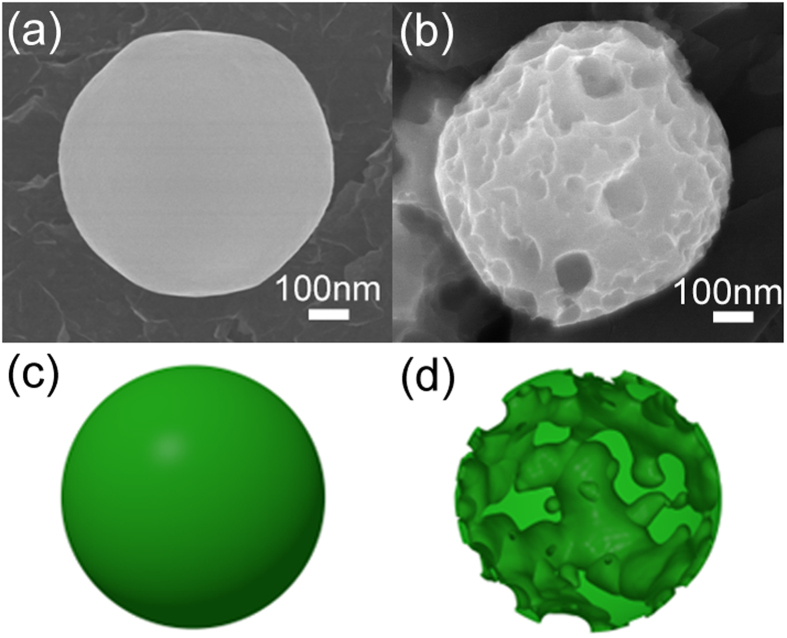
GeO_2_ dissolution in water over time. SEM morphologies of Ge SMS freshly synthesized by LML in water (**a**) and porous SMS formed after storage in water for 1 week due to GeO_2_ dissolution. (**c**) and (**d**) depict sketches of (**a**) and (**b**).

Milled Ge educt powders (Fig. 1a–c) naturally cover a large size range from 30 nm to 20 μm. In the following, the size classes of <200 nm (fine fraction), 100–1000 nm (hydrodynamic fraction), and 1–20 μm (microfraction) are discussed separately. Figure 1f,g show the TEM images of the LML-synthesized Ge SMSs prepared in water (Fig. 1f) and isopropanol (Fig. 1g) after 100 liquid jet passages using the experimental setup shown in Fig. 1d which was developed by Lau *et al*.^46^ and is based on the idea of Wagener *et al*.^47^ who utilized a free liquid jet for laser irradiation of particles. XRD analysis (Fig. 1e) shows that the Ge SMSs have a crystalline structure with five peaks located at 27.23°, 45.31°, 53.65°, 65.97° and 72.77° corresponding to the (111), (220), (311), (400) and (331) of the Fd-3mS (227) structure of Ge (ICSD-43422), respectively. The calculated crystal domain diameter sizes for the educt Ge powder, the Ge SMSs obtained in water, and the Ge SMSs synthesized in isopropanol are 25.4 nm, 32.3 nm and 36.0 nm, respectively. The smaller crystalline sizes calculated from XRD compared to SMSs’ size might be caused by polycrystallinity of the Ge SMSs. There is a peak shoulder at an angle of 27° (Fig. 1e), which is also corresponds to the peak of GeO_2_ (ICSD-43422). Because these two peaks, to some extent, overlap with each other, we cannot exclude the existence of GeO_2_. But from the dissolution test shown in Fig. 2, the presence of a GeO_2_ shell which encapsulates the Ge core is corroborated. Similarly, oxidative reactions have been observed during LML for TiN, leading to the formation of TiN, TiO_2_, and TiOxNy^48^. It is well known that Ge and GeO_2_ have different solubility in water. GeO_2_ is easy to be dissolved in water following the reaction of 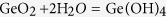
49, whereas Ge is insoluble in water^50^. To test whether the Ge SMSs are partially oxidized, some Ge SMSs synthesized by LML in water were preserved in water and taken out for SEM measurement after one week immersion time. As shown in Fig. 2, the Ge SMSs become porous, indicating the existence of GeO_2_ on the surface of the Ge SMSs. Therefore, GeO_2_ and Ge apparently coexist in the SMSs at least after storage in water.

**Figure 3:**
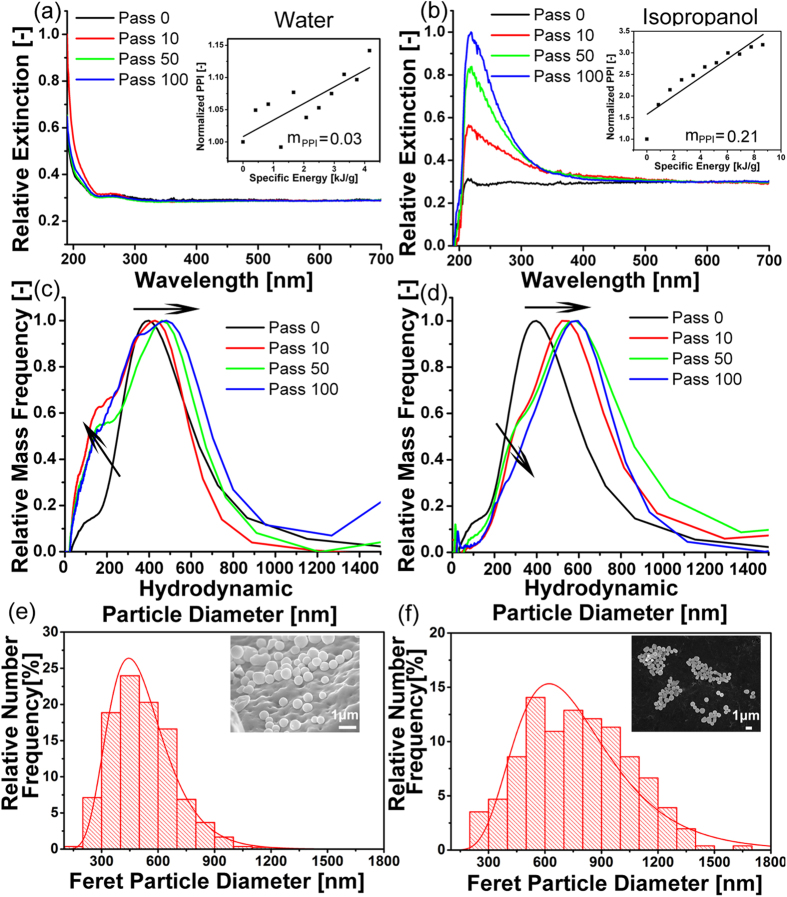
(**a,b**) UV-vis extinction spectra and (**c,d**) size distribution of the irradiated Ge colloid after increasing number of jet passages in water (**a,c**) and isopropanol (**b,d**), respectively. Black-0 passage; Red-10 passages; Green-50 passages; Blue-100 passages. PPI values as a function of specific energy from 0 to 100 in 10 passages increments for both liquids are shown as the inset figures in Fig. 2(a,b). One passage equals 0.42 J/g and 0.86 J/g laser energy input of water and isopropanol, respectively. (**e,f**) Histograms showing Feret diameter of particles before and after laser melting. The relationship between relative mass frequency and hydrodynamic particle diameter as shown in Fig. 3c,d was extracted from analytical disc centrifuge measurements.

Figure 3a and b show the UV-vis extinction spectra of the Ge colloids after 0, 10, 50 and 100 passages in water and isopropanol, respectively. As presented in our previous works^46^,^51^, a primary particle index (PPI) deduced from the UV–vis spectra is a good indication of whether the particle size is reduced or enlarged. It is a value obtained by dividing the relative extinction maximum to the extinction value at 600 nm. To make the PPI result more obvious for Ge SMSs, all spectra are normalized at 600 nm. The PPI values calculated for passages from 0 to 100 in 10 passages increments for both liquids are shown as insets in Fig. 3a,b. It is evident that the maximal absorption peak intensity increases and scattering decreases with increasing liquid jet passage number, indicating a reduction of scattering objects to form SMSs. Compared with the trend in water (Fig. 3a), the change of the maximal absorption peak intensity is much clearer in isopropanol (Fig. 3b). This can also be deduced from the higher m_PPI_ value^46^ of 0.21 in isopropanol than that of 0.03 in water.

To confirm the size increase behavior of Ge particles deduced from the red-shift of UV-vis spectra in Fig. 3b, the particles’ size distribution on the nanoscale is characterized by ADC for colloids obtained after 10, 50 and 100 passages (Fig. 3c,d). Both ADC and SEM data demonstrate that the majority of the educt is in the range of 0–400 nm. But there is a great disparity between the size measurements of SEM and ADC for educts ≤200 nm. We attribute this to our observation that small educts (≤200 nm) are much easier attaching to the microscale educt in consequence of forces acting between these particles. This will make an ADC detection difficult. As clearly seen, the average size (mass-weighted hydrodynamic diameter) of the Ge educts is ~400 nm and it gradually increases as the LML proceeds. Compared with the Ge SMSs synthesized in water (Fig. 3c) that reach sizes of ~500 nm after 100 passages (and the size fraction below 200 nm increases), the Ge SMSs synthesized in isopropanol (Fig. 3d) after 100 passages are a slightly bigger, about 600 nm in diameter (and size fraction below 200 nm decreases). This is in agreement with the size distribution calculated from SEM images (Fig. 1e,f). The size disparity of Ge SMSs in water and isopropanol is attributed to the higher specific energy input for the educts in isopropanol compared to water (as the higher viscous isopropanol flows slower through the passage reactor). This is in accordance with trend that higher laser fluences produce larger SMSs^29^ and that the smaller educt fraction (<100 nm) is consumed by LML-induced aggregation reshaping. Therefore, from both UV-Vis and ADC results, it can be concluded that a simultaneous bi-directional (melting and fragmentation) particle transformation occurs during ps laser irradiation of Ge particles in water, which is different from previous findings where either ps laser fragmentation or laser melting happens^46^ at defined laser fluence. In isopropanol, the average hydrodynamic diameter is increased, and fraction of particles <200 nm is decreased. The reason for this interesting phenomenon will be discussed later.

Due to the longer electron-phonon coupling period of the excited material compared to the pulse duration, fs laser ablation is generally considered as a “cold” ablation process where Coulomb explosion dominates the fragmentation process of nanoparticle educts^52^. Ns lasers, in turn, are extensively adopted for the LML-synthesis of SMSs^27^,^28^,^44^ since within nanoseconds the energy-transfer from electron to lattice can take place to allow both electron and lattice to be in thermal equilibrium^52^. Considering, that the transition between cold and hot ablation is in the picosecond regime, there should be a possibility to melt materials using ps lasers if the pulse duration is longer than the electron-lattice coupling period of the irradiated material. Roskos *et al*. reported that hot electrons in Ge cool down within 5 ps^53^. This suggests that ps lasers with pulse duration longer than 5 ps are able to melt Ge particles, which has been proven by Siegel *et al*. who showed the melting behavior of a germanium film using a 30 ps laser^54^. Therefore, the 10 ps laser utilized in our experiments should also be appropriate to melt Ge powders and transform them into SMSs. An interest in ps-LML might be related to the capability to finely tune the melting process. Ps-pulses typically have low pulse energies for the irradiation of large volumes. Since the pulse duration is in between cold (fs) ablation and hot (ns) ablation, it is possible to gradually melt the particles by adjusting the energy dose. A further benefit might be related to upscaling. At the same laser power, conventional ps laser systems usually have higher repetition rates than ns lasers due to technical reasons related to the higher peak power of pulses with shorter pulse durations. Higher repetition rates, in turn, increase the amount of pulses interacting with a single particle in the liquid jet and lead to a faster processing. Similar to incubation effects observed during LAL with MHz lasers^5^,^6^, high-repetition rate LML might also benefit from incubation.

**Figure 4:**
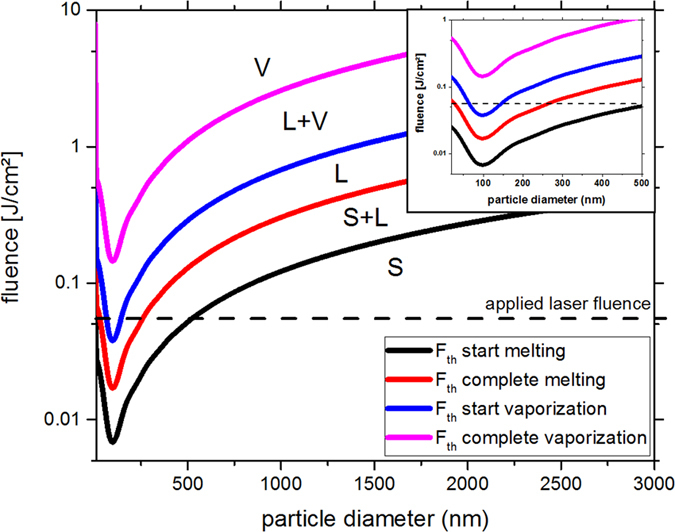
Calculated fluence thresholds (F_th_) showing the different particle states solid (s), liquid (l) and vaporized (v) in dependence of their size according to the model developed by Pyatenko*et al*.^44^ (neglecting the heat loss). The dotted line marks the laser fluence that the particles are irradiated with during each passage. Inset shows a magnification of the diameter range 10–500 nm.

To further analyze the melting behavior of the Ge powders under our experimental conditions, the fluence thresholds for melting and vaporization of the particles in dependence of their size were calculated using the model developed by Pyatenko *et al*.^30^. The results are shown in Fig. 4. Note that we neglect the heat loss, as we use ps pulses and assume spherical particles. The dotted line indicates the applied laser fluence during particle irradiation for each passage. Figure 4 shows that the laser fluence used for particle irradiation theoretically addresses particle melting for particles smaller than ~540 nm, while the particles larger ~50 nm and smaller ~140 nm should be vaporized. However, we experimentally obtained SMSs larger than 540 nm and observed a gradual size increase for 50~140 nm educt (Fig. 3c,d), indicating that further factors should be taken into account during SMS formation besides thermal equilibrium melting process of spherical structures. To understand how the educt particles evolve into SMSs, four size-dependent particle formation mechanisms will be discussed in the following part. Overall, due to the potential heat coupling within a few ps in Ge particles we believe that 10 ps pulses are appropriate to initialize Ge powder/particle melting and the determination of these thresholds strongly supports this conclusion. Hence, the feasibility to use a ps laser to produce SMSs via pulsed LML is demonstrated experimentally and theoretically using a picosecond laser with pulse duration allowing electron-phonon coupling of Ge upon laser irradiation.

## Discussion

**Figure 5:**
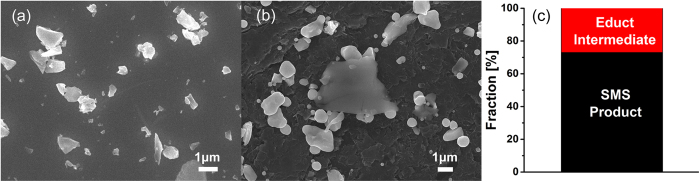
(**a**) SEM images of the Ge educt powder. (**b**) SEM image showing the particles after 10 passages of ps-laser irradiation in water. Educt particles, as well as intermediates and SMS products are apparent. (**c**) Diagram showing the fraction between educt & intermediate and SMS product after 10 passages of ps-laser irradiation in water.

A scenario for SMSs formation during ns LML have been proposed by Koshizaki and co-workers^55^,^56^. To artificially decrease the NPs’ distance and to increase the chance for adjacent particles to interact and merge into SMSs upon laser irradiation, NPs agglomeration or aggregation is considered to be a prerequisite step for SMSs formation from nanoparticle educts. In general, NP aggregation is mostly realized by salts (e.g., NaCl) during sample preparation or by adding stabilizing reagent (e.g., citrate) during the LML process^36^. Despite successful synthesis of a series of SMSs, the embedment or integration of additives inside the SMSs may increase the risk of contamination and compromise the function performance for desirable applications, such as catalysis, solar cell, etc. Therefore, a cleaner LML synthesis process is much desirable. To this end, the experiments presented here took use of well-dispersed powder educts without any additives (e.g. ions, ligands, surfactants, etc.). This aroused the interest of isochorically reshaping powders into SMSs without the need of aggregation^57^. Since the Ge educts are obtained from milling of the Ge wafers, the mechanical crack of the Ge wafers gives rise to raw powders characterized by sharp edges (Fig. 5a). In the following section, we discuss four size dependent possible mechanisms for educts to transform into SMSs.

Insights regarding the formation mechanism can be gained from few liquid passages since limited SMSs quantity is beneficial to reconstruct the initial melting behavior of the educts. As reported previously, a liquid jet reactor avoids any back-mixing so that intermediates of LML process are captured after a defined number of passages^46^,^58^. After 10 passages, ~70% of small educts with sizes from 200~1000 nm are transformed into SMSs as depicted in Fig. 5b,c. This is different from the theoretical prediction in Fig. 4 which shows that only educts with sizes less than 540 nm but larger than 140 nm could be melted. A first possible particle formation mechanism for the direct educt transformation into SMSs is isochoric laser reshaping below the material melting temperature. This phenomenon has been experimentally observed and confirmed by many authors. For example, Inasawa *et al*. reported that gold nanorods could be reshaped into nanospheres when the temperature is 120 K lower than the melting point (1337 K)^59^. Petrova showed that a temperature of 523 K could also trigger the transformation of gold nanorods to nanospheres^60^ and that the temperature threshold for Au nanorods’ reshaping is as low as 373 K^60^. These works indicate that LML-induced material reshaping does not require the materials to reach the bulk material melting point, such as the melting point (1210 K) of Ge. As a result, educts with sizes in the range of 200~1000 nm can be directly reshaped into SMSs, similar to the previously reported LML-induced shape conversion^57^. The driving force of shape conversion from irregular powders to spherical particles is due to its smallest surface area among all surfaces at a given volume, that is, smallest surface energy for spherical particles^61^. Please note the limitation of the model used to create Fig. 4; reports such as ref. 37 have shown that the real fluence threshold is typically lower than the calculated threshold due to following two reasons. When a Gaussian energy profile is used for irradiation, the peak fluence of the beam is higher than the values obtained by integrating the intensity profile. Also, the assumption of spherical educt particles is not applicable to our educt particles as can be clearly seen in Fig. 5a.

**Figure 6:**
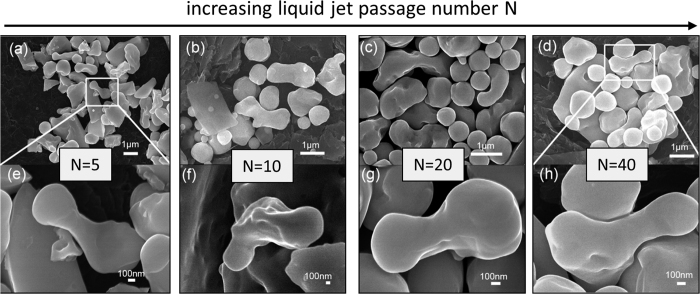
(**a–d**) SEM images of the Ge particles and structures after 5, 10, 20, 40 passages of laser irradiation in water, respectively, showing the gradual increase of number of SMS with increasing passage number (**e–h**) SEM images of anisotropic structures obtained after 5, 10, 20, 40 passages of laser irradiation in water, respectively, showing that these anisotropic structures can be found after each passage, indicating the bisection mechanism.

The second possible SMS formation mechanism we observe is the bisection of instable (oscillating) droplets^62^ into smaller SMSs due to the reshaping of larger particles with a dimensions of 1000–2000 nm (Fig. 6e–h). Similar to direct reshaping of the educts with sizes of 200~1000 nm, the educts first evolve into anisotropic structures during the LML. Such anisotropic particles with aspect ratios of 2–4 and the size of the narrow middle parts being 200~300 nm appear after 5, 10, 20 and 40 passages (Fig. 6a–d). Upon subsequent laser irradiation, these anisotropic structures are carved and split into separate SMSs, following the bisection mechanism proposed by Kuzmin *et al*.^62^,^63^. This reshaping-carving-split bisection process is assumed to start at the beginning of LML since the anisotropic structures are found already after 5 passages. This process will continue as LML proceeds; that is why we can still find these anisotropic structures after 100 passages.

**Figure 7:**
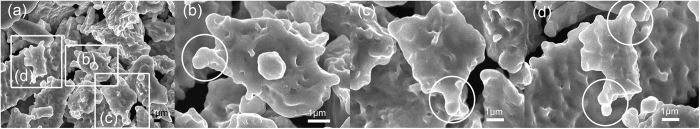
(**a**) Overview SEM image of the LML-produced microstructures after 40 passages. (**b**–**d**) Magnified SEM images of three microstructures characterized with many spherical tips (as marked by white circles) and molten traces. Separation of these spherical tips from microstructures leads to the formation of SMSs.

The third SMSs formation mechanism is assumed to originate from the educt fraction with sizes larger than 2000 nm. Possibly, a melting-detachment mixed mechanism leads to the formation of SMSs from these relatively large particles. This assumption is derived from experimental observations between the educts (see Fig. 1a,b) and the products (Fig. 7). As shown in Fig. 7, the surfaces of educts with sizes larger than 2000 nm were melted by 40 passages of ps laser irradiation. Interestingly, these molten structures are featured by spherical tips which seem to split out of the molten surface structures, similar to the case of bisection mechanism shown in Fig. 6. The split of smaller pieces (spherical tips) can be caused by laser-induced shock waves. These detached fragments are good candidates to be further reshaped into spheres by subsequent passages. The time of detachment of these small pieces from the melted structures and the amount of detachment will be studied in a further study and are not in the scope of this paper. Complete melting of large educt with sizes larger than 2000 nm may be possible but will require a huge amount of passages’ irradiation or higher laser pulse energy. This problem has been reported by the Koshizaki group^34^ who found that LML efficiency for large Si powders (several micrometers) without milling was far lower compared with LML of small educt powders obtained after milling (30 mins’ irradiation with laser fluence of 460 mJ/cm^2^).

The fourth SMSs formation mechanism is related to the educts smaller than 200 nm because of their gradual size increase as indicated in Fig. 3c,d. For their growth into bigger SMSs, the well-known agglomeration-induced LML^36^,^37^ is the preferable route. This finding is in accordance with the reports by Serkov *et al*. who achieved nanoparticles agglomeration by ps laser irradiation of disperse Ag and Au colloidal mixtures^64^ and Mafuné *et al*. who obtained “nanoweb” consisting of many nanoparticles after laser irradiation of a mixture of disperse Au and Pt NPs^65^. The formation of agglomerates could be further stimulated by nanobubble collapse and its induced shock waves. The smaller particle fraction of the educt may encounter each other and then be melted into SMSs by the ps laser irradiation. The enlargement of LAL-synthesized particle into SMSs shown here seems to contradict with Fig. 4 which shows that particles with sizes in the range of 50~140 nm should undergo evaporation upon laser irradiation. However, as noted before we assume that the real fluence required to induce PLML is lower as predicted by the model used in Fig. 4.

**Figure 8:**
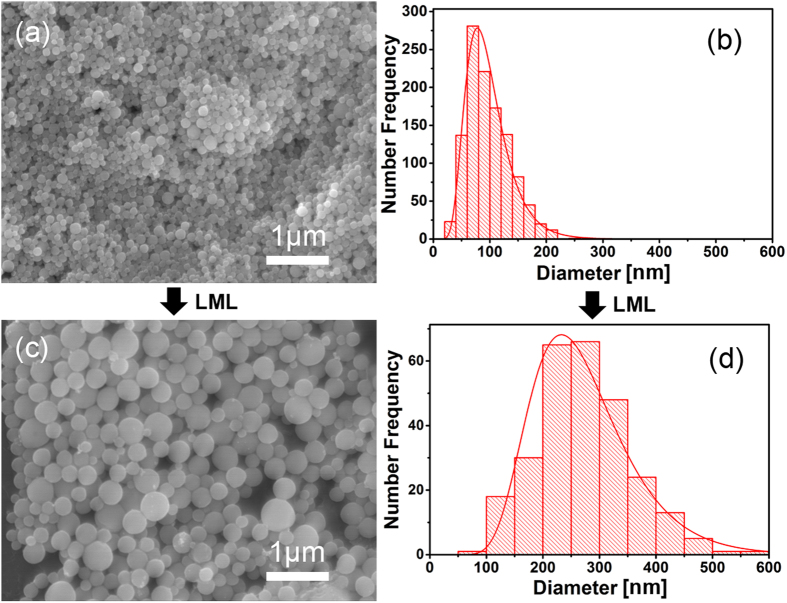
(**a,b**) SEM morphologies and size distribution of Ge nanoparticles obtained by laser ablation in water. (**c,d**) SEM morphologies and size distribution of Ge SMSs synthesized by post laser irradiation (with 50 passages) of the LAL-synthesized particles.

In order to corroborate our hypothesis that small educts could evolve into bigger SMSs during laser irradiation, we first synthesized Ge particles by LAL in water (10–100 nm, peak at 80 nm). Just after the nanoparticles synthesis, they were directly transferred to the LML setup for ps laser irradiation so that the aging-induced agglomeration of the synthesized particles is minimized. The morphologies and sizes of both of the LAL-synthesized Ge nanoparticles and nanoproducts of their post-irradiation by LML are compared, as shown in Fig. 8. As clearly shown, small Ge NPs (~80 nm in diameter) synthesized by laser ablation (Fig. 8a,b) were transformed into significantly bigger Ge SMSs (~230 nm in diameter) after 50 passages (Fig. 8c,d), hence demonstrating the feasibility of small Ge particles to become larger SMSs by ps LML, which also agrees well with the gradual size increase of small Ge particles towards larger SMSs shown in Fig. 2c,d.

**Figure 9:**
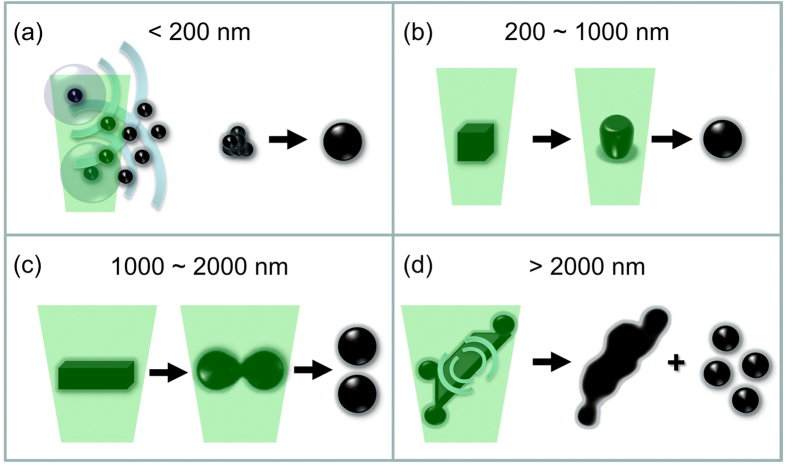
The four educt size dependent SMS formation mechanism in the range of (**a**) <200 nm (aggregation-melting), (**b**) 200~1000 nm (reshaping), (**c**) 1000~2000 nm (bisectioning) and (**d**) >2000 nm (surface melting, detachment).

Overall, four size-dependent mechanism contribute to the Ge SMS formation, as schematically depicted in Fig. 9. When the educt is less than 200 nm, they will aggregate by laser heating or laser-induced shockwaves and then melted into SMSs (Fig. 9a). Educts with sizes in the range of 200~1000 nm are directly reshaped into SMSs (Fig. 9b). Larger educts (>1000 nm) tend to be reshaped and split by ps laser irradiation (Fig. 9d). Educts with sizes between 1000 and 2000 nm undergo melting, bisection and reshaping processes to evolve into SMSs, while some fragments that are split from educts larger than 2000 nm act as precursors for the SMSs (Fig. 9c).

## Conclusions

In conclusion, we have demonstrated the feasibility to obtain Ge SMSs using ps laser irradiation of germanium powders containing both small (nanoscale) and large (microscale) particles. From the analysis of UV-Vis spectra, ADC data and SEM images showing the particles’ evolution after different passages upon laser irradiation, four SMSs formation mechanisms for different scale educts are proposed. Direct melting-reshaping is the favored route for the educts (powders) with diameters in the range of 200~1000 nm to be transformed isochorically into SMSs, while the transformation of relative bigger powders (1000~2000 nm) into SMSs follows the procedures of melting, splitting (bisectioning) and spherization. As for educts larger than 2000 nm, cavitation bubble-collapse induced shock waves may contribute to the melting of educt’s surface into molten structures with many tips whose separation from the structures are good candidates for SMS formation upon further laser irradiation, while with regard to small educts less than 200 nm in size, the particles are aggregated and molten into SMSs. We assume that the counterintuitive melting process by picosecond laser irradiation takes place when the laser pulse duration (10 ps) is longer than the electron-phonon coupling period of the irradiated material (5 ps for Ge). Given that this constant is in the ps regime for many materials (e.g., Au: 3–4 ps^66^; Cu: 1–4 ps^67^; Si: 0.4 ps^68^; ZnO: 0.5 ps^69^), it is anticipated that ps LML-induced SMSs transformation is also applicable to further metals and metal oxides.

## Methods

The experimental setup for ps laser fragmention in confined liquid flow has been described previously^46^,^47^,^58^. A liquid passage reactor connected with a reservoir with diameter of 1.3 mm is used for our experiments. The generated suspension filament, namely liquid jet, is irradiated by a laser beam with defined laser fluences. 50 ml colloidal solutions with Ge powder mass concentration of 1 g/L in water and isopropanol are used, respectively. Ge powders are milled from Ge wafers with a thickness of 150 μm and consisting of 94.38wt.% of germanium, 1wt.% of oxygen, 4.33wt.% of carbon, 0.29wt.% of aluminum. The Ge powders are pre-treated in an ultra-sonication bath prior to laser irradiation as proposed by Blandin *et al*.^70^. This pre-treatment is crucial since it is difficult to induce an effect by laser irradiation on very large raw particles, as pointed out in the Li X. *et al*.^34^. A ps laser (Ekspla, atlantic series) is employed with pulse duration of 10 ps, pulse energy of 75 μJ at 532 nm and a repetition rate of 100 kHz. The distance between the center of the liquid filament and the lens (100 mm focal length) is set to be 88 mm resulting in laser fluence of 0.06 J/cm^2^ at the filament’s surface for LML of Ge powders. Due to the difference of liquid viscosity (water-0.89mPa·s; isopropanol-2.1 mPa·s), one passage period for colloids in water and isopropanol is 26 s and 36 s, respectively. As for laser ablation, a germanium wafer is immersed inside a glass chamber with 30 ml water and liquid thickness of 3 mm above the target. Afterwards, the target is ablated for ten minutes by a ps laser with a pulse energy of 80 μJ, a wavelength of 1064 nm, a pulse duration of 10 ps, a pulse energy of 75 μJ and a repetition rate of 10 kHz. The colloidal solution is then transferred to the LML setup and irradiated using the same parameters as LML of Ge powders for 50 passages. In a final step, the Ge NPs obtained from both laser ablation and post-irradiation are centrifuged at 4000 rounds/min for half an hour and characterized by scanning electron microscopy (SEM) operated at 15 kV and equipped with an EDS. Size distribution of the microspheres at different laser fluences is analyzed with the *ImageJ* software. Time-resolved transmission electron microscopy (TEM, Phillips, CM12) and X-ray diffraction (X-Pert PRO) with Ni-filtered Cu Kα (0.154 nm) radiation detected by an X-Celerator detector are used to characterize the Ge SMSs morphology and crystal structure, respectively.

## Additional Information

**How to cite this article**: Zhang, D. *et al*. Germanium Sub-Microspheres Synthesized by Picosecond Pulsed Laser Melting in Liquids: Educt Size Effects. *Sci. Rep.* **7**, 40355; doi: 10.1038/srep40355 (2017).

**Publisher's note:** Springer Nature remains neutral with regard to jurisdictional claims in published maps and institutional affiliations.

## References

[b1] Fojtik A. & Henglein A. Laser ablation of films and suspended particles in a solvent: formation of cluster and colloid solutions. Ber. Bunsenges. Phys. Chem. 97, 252–254 (1993).

[b2] Neddersen J., Chumanov G. & Cotton T. M. Laser ablation of metals: a new method for preparing SERS active colloids. Appl. Spectrosc. 47, 1959–1964 (1993).

[b3] Mafuné F., Kohno J.-y., Takeda Y., Kondow T. & Sawabe H. Formation and size control of silver nanoparticles by laser ablation in aqueous solution. The Journal of Physical Chemistry B 104, 9111–9117 (2000).

[b4] Mafuné F., Kohno J.-y., Takeda Y. & Kondow T. Full Physical Preparation of Size-Selected Gold Nanoparticles in Solution: Laser Ablation and Laser-Induced Size Control. The Journal of Physical Chemistry B 106, 7575–7577 (2002).

[b5] Streubel R., Barcikowski S. & Gökce B. Continuous multigram nanoparticle synthesis by high-power, high-repetition-rate ultrafast laser ablation in liquids. Opt. Lett. 41, 1486–1489 (2016). 27192268 10.1364/OL.41.001486

[b6] Streubel R., Bendt G. & Gökce B. Pilot-scale synthesis of metal nanoparticles by high-speed pulsed laser ablation in liquids. Nanotechnology 27, 205602 (2016). 27053598 10.1088/0957-4484/27/20/205602

[b7] Zhang D., Gökce B., Notthoff C. & Barcikowski S. Layered Seed-Growth of AgGe Football-like Microspheres via Precursor-Free Picosecond Laser Synthesis in Water. Scientific reports 5, 13661 (2015). 26334136 10.1038/srep13661 PMC4558578

[b8] Castro H. P. . Synthesis and characterisation of fluorescent carbon nanodots produced in ionic liquids by laser ablation. Chem.–Eur. J. 22, 138–143 (2015). 26558445 10.1002/chem.201503286

[b9] Amendola V. & Meneghetti M. What controls the composition and the structure of nanomaterials generated by laser ablation in liquid solution? Physical Chemistry Chemical Physics 15, 3027–3046 (2013). 23165724 10.1039/c2cp42895d

[b10] Nguyen V., Si J., Yan L. & Hou X. Electron-hole Recombination Dynamics in Carbon Nanodots. Carbon (2015).

[b11] Luo N. . Ligand-free gadolinium oxide for *in vivo* T1-weighted magnetic resonance imaging. Physical Chemistry Chemical Physics 15, 12235–12240, doi: 10.1039/c3cp51530c (2013). 23771105

[b12] Amendola V., Riello P. & Meneghetti M. Magnetic nanoparticles of iron carbide, iron oxide, iron@ iron oxide, and metal iron synthesized by laser ablation in organic solvents. The Journal of Physical Chemistry C 115, 5140–5146 (2011).

[b13] Zhang D. . Formation mechanism of laser-synthesized iron-manganese alloy nanoparticles, manganese oxide nanosheets and nanofibers. *Part. Part. Syst. Charact.*, doi: 10.1002/ppsc.201600225 (2016).

[b14] Ishikawa Y., Kawaguchi K., Shimizu Y., Sasaki T. & Koshizaki N. Preparation of Fe–Pt alloy particles by pulsed laser ablation in liquid medium. Chemical physics letters 428, 426–429 (2006).

[b15] Zhang J. . Preparation of PtAu alloy colloids by laser ablation in solution and their characterization. The Journal of Physical Chemistry C 116, 13413–13420 (2012).

[b16] Zhang D. & Gökce B. Perspective of laser-prototyping nanoparticle-polymer composites. Appl. Surf. Sci. 392, 991–1003 (2017).

[b17] Schmitz C., Gökce B., Jakobi J., Barcikowski S. & Strehmel B. Integration of Gold Nanoparticles into NIR-Radiation Curable Powder Resin. ChemistrySelect 1, 5574–5578 (2016).

[b18] Jonusauskas L. . Plasmon assisted 3D microstructuring of gold nanoparticle-doped polymers. Nanotechnology 27, 154001 (2016). 26925538 10.1088/0957-4484/27/15/154001

[b19] Wang H., Odawara O. & Wada H. Facile and chemically pure preparation of YVO_4_: Eu^3+^ colloid with novel nanostructure via laser ablation in water. Sci. Rep. 6, 20507 (2016). 26842419 10.1038/srep20507 PMC4740808

[b20] Liang Y. . A microfibre assembly of an iron-carbon composite with giant magnetisation. Scientific reports 3, 3051 (2013). 24165864 10.1038/srep03051 PMC3810667

[b21] Barcikowski S., Baranowski T., Durmus Y., Wiedwald U. & Gökce B. Solid solution magnetic FeNi nanostrand–polymer composites by connecting-coarsening assembly. J. Mater. Chem. C 3, 10699–10704 (2015).

[b22] Kamat P. V. Photophysical, photochemical and photocatalytic aspects of metal nanoparticles. J. Phys. Chem. B 106, 7729–7744 (2002).

[b23] Werner D., Furube A., Okamoto T. & Hashimoto S. Femtosecond Laser-Induced Size Reduction of Aqueous Gold Nanoparticles: *In Situ* and Pump− Probe Spectroscopy Investigations Revealing Coulomb Explosion. The Journal of Physical Chemistry C 115, 8503–8512 (2011).

[b24] Werner D., Ueki T. & Hashimoto S. Methodological Improvement in Pulsed Laser-Induced Size Reduction of Aqueous Colloidal Gold Nanoparticles by Applying High Pressure. The Journal of Physical Chemistry C 116, 5482–5491 (2012).

[b25] Werner D. & Hashimoto S. Controlling the Pulsed-Laser-Induced Size Reduction of Au and Ag Nanoparticles via Changes in the External Pressure, Laser Intensity, and Excitation Wavelength. Langmuir 29, 1295–1302 (2013). 23259708 10.1021/la3046143

[b26] Boyer P., Ménard D. & Meunier M. Nanoclustered Co− Au Particles Fabricated by Femtosecond Laser Fragmentation in Liquids. The Journal of Physical Chemistry C 114, 13497–13500 (2010).

[b27] Wang H., Pyatenko A., Koshizaki N., Moehwald H. & Shchukin, D. Single-Crystalline ZnO Spherical Particles by Pulsed Laser Irradiation of Colloidal Nanoparticles for Ultraviolet Photodetection. ACS Applied Materials & Interfaces 6, 2241–2247 (2014). 24533659 10.1021/am500443a

[b28] Wang H. . Size‐Tailored ZnO Submicrometer Spheres: Bottom‐Up Construction, Size‐Related Optical Extinction, and Selective Aniline Trapping. Advanced Materials 23, 1865–1870 (2011). 21413086 10.1002/adma.201100078

[b29] Wang H. . Selective pulsed heating for the synthesis of semiconductor and metal submicrometer spheres. Angewandte Chemie 122, 6505–6508 (2010). 10.1002/anie.201002963 20677301

[b30] Hunter B. M. . Highly Active Mixed-Metal Nanosheet Water Oxidation Catalysts Made by Pulsed-Laser Ablation in Liquids. Journal of the American Chemical Society 136, 13118–13121 (2014). 25197774 10.1021/ja506087h

[b31] Kymakis E. . Plasmonic bulk heterojunction solar cells: the role of nanoparticle ligand coating. ACS Photonics 2, 714–723 (2015).

[b32] Amendola V. . Magneto-Plasmonic Au-Fe Alloy Nanoparticles Designed for Multimodal SERS-MRI-CT Imaging. Small 10, 2476–2486 (2014). 24619736 10.1002/smll.201303372

[b33] Kabashin A. V. & Timoshenko V. Y. What theranostic applications could ultrapure laser-synthesized Si nanoparticles have in cancer? Nanomedicine 11, 2247–2250 (2016). 27527580 10.2217/nnm-2016-0228

[b34] Li X. . Fabrication of crystalline silicon spheres by selective laser heating in liquid medium. Langmuir 27, 5076–5080 (2011). 21413711 10.1021/la200231f

[b35] Tsuji T. . Preparation and investigation of the formation mechanism of submicron-sized spherical particles of gold using laser ablation and laser irradiation in liquids. Physical Chemistry Chemical Physics 15, 3099–3107 (2013). 23303286 10.1039/c2cp44159d

[b36] Tsuji T., Higashi Y., Tsuji M., Ishikawa Y. & Koshizaki N. Preparation of submicron-sized spherical particles of gold using laser-induced melting in liquids and low-toxic stabilizing reagent. Applied Surface Science 348, 10–15, (2015).

[b37] Rehbock C., Zwartscholten J. & Barcikowski S. Biocompatible gold sub micrometer spheres with variable surface texture fabricated by Pulsed Laser Melting in Liquid (PLML). Chemistry Letters 43, 1502–1504 (2014).

[b38] Ishikawa Y., Feng Q. & Koshizaki N. Growth fusion of submicron spherical boron carbide particles by repetitive pulsed laser irradiation in liquid media. Applied Physics A 99, 797–803 (2010).

[b39] Li X. . Preparation of silver spheres by selective laser heating in silver-containing precursor solution. Optics express 19, 2846–2851 (2011). 21369105 10.1364/OE.19.002846

[b40] Wang H. . General Bottom‐Up Construction of Spherical Particles by Pulsed Laser Irradiation of Colloidal Nanoparticles: A Case Study on CuO. Chemistry-A European Journal 18, 163–169 (2012). 10.1002/chem.201102079 22140012

[b41] Hu X. . Laser-induced reshaping of particles aiming at energy-saving applications. J. Mater. Chem. 22, 15947–15952 (2012).

[b42] Okamoto S. . Fabrication of single-crystalline microspheres with high sphericity from anisotropic materials. Scientific Reports 4, 5186 (2014). 24898213 10.1038/srep05186 PMC4046134

[b43] Wang H. . Single-crystalline rutile TiO2 hollow spheres: room-temperature synthesis, tailored visible-light-extinction, and effective scattering layer for quantum dot-sensitized solar cells. Journal of the American Chemical Society 133, 19102–19109 (2011). 22017378 10.1021/ja2049463

[b44] Pyatenko A., Wang H., Koshizaki N. & Tsuji T. Mechanism of pulse laser interaction with colloidal nanoparticles. Laser & Photonics Reviews 7, 596–604 (2013).

[b45] Koshizaki N. In4th conference on advanced nanoparticle generation and excitation by lasers in liquids (angel) (Essen, 2016).

[b46] Lau M. & Barcikowski S. Quantification of mass-specific laser energy input converted into particle properties during picosecond pulsed laser fragmentation of zinc oxide and boron carbide in liquids. Applied Surface Science 348, 22–29 (2015).

[b47] Wagener P. & Barcikowski S. Laser fragmentation of organic microparticles into colloidal nanoparticles in a free liquid jet. Applied Physics A 101, 435–439 (2010).

[b48] Kawasoe K. . Preparation of spherical particles by laser melting in liquid using TiN as a raw material. Appl. Phys. B 119, 475–483 (2015).

[b49] Pokrovski G. S. & Schott J. Thermodynamic properties of aqueous Ge (IV) hydroxide complexes from 25 to 350 C: implications for the behavior of germanium and the Ge/Si ratio in hydrothermal fluids. Geochim. Cosmochim. Acta 62, 1631–1642 (1998).

[b50] Weast R. C. Handbook of Chemistry and Physics. 70th. Boc Raton : CRC Press Inc (1989).

[b51] Rehbock C., Merk V., Gamrad L., Streubel R. & Barcikowski S. Size control of laser-fabricated surfactant-free gold nanoparticles with highly diluted electrolytes and their subsequent bioconjugation. Physical Chemistry Chemical Physics 15, 3057–3067 (2013). 23132176 10.1039/c2cp42641b

[b52] Chichkov B., Momma C., Nolte S., Von Alvensleben F. & Tünnermann A. Femtosecond, picosecond and nanosecond laser ablation of solids. Applied Physics A 63, 109–115 (1996).

[b53] Roskos H., Rieck B., Seilmeier A. & Kaiser W. Cooling of a carrier plasma in germanium investigated with subpicosecond infrared pulses. Appl. Phys. Lett. 53, 2406–2408 (1988).

[b54] Siegel J., Solis J. & Afonso C. N. Recalescence after solidification in Ge films melted by picosecond laser pulses. Applied Physics Letters 75, 1071–1073 (1999).

[b55] Pyatenko A., Wang H. & Koshizaki N. Growth Mechanism of Monodisperse Spherical Particles under Nanosecond Pulsed Laser Irradiation. The Journal of Physical Chemistry C 118, 4495–4500 (2014).

[b56] Tsuji T. . Fabrication of Spherical-Shaped Submicron Particles of ZnO Using Laser-induced Melting of Submicron-sized Source Materials. Journal of Laser Micro/Nanoengineering 8 (2013).

[b57] Liu D. . Rapid Synthesis of Monodisperse Au Nanospheres through a Laser Irradiation-Induced Shape Conversion, Self-Assembly and Their Electromagnetic Coupling SERS Enhancement. Scientific reports 5, 7686 (2015). 25566872 10.1038/srep07686 PMC4286736

[b58] Lau M., Ziefuss A., Komossa T. & Barcikowski S. Inclusion of supported gold nanoparticles into their semiconductor support. Phys. Chem. Chem. Phys. 17, 29311–29318 (2015). 26467473 10.1039/c5cp04296h

[b59] Inasawa S., Sugiyama M. & Yamaguchi Y. Laser-induced shape transformation of gold nanoparticles below the melting point: the effect of surface melting. J. Phys. Chem. B 109, 3104–3111 (2005). 16851329 10.1021/jp045167j

[b60] Petrova H. . On the temperature stability of gold nanorods: comparison between thermal and ultrafast laser-induced heating. Phys. Chem. Chem. Phys. 8, 814–821 (2006). 16482322 10.1039/b514644e

[b61] Song X. . Submicron-Lubricant Based on Crystallized Fe3O4 Spheres for Enhanced Tribology Performance. Chemistry of Materials 26, 5113–5119 (2014).

[b62] Kuzmin P., Shafeev G., Serkov A., Kirichenko N. & Shcherbina M. Laser-assisted fragmentation of Al particles suspended in liquid. Applied Surface Science 294, 15–19 (2014).

[b63] Kirichenko N. A., Shcherbina M. E., Serkov A. A. & Rakov I. I. Transfer equation for the description of the dynamics of Au nanoparticle ensemble in liquid under pulsed laser irradiation. Appl. Surf. Sci. 376, 180–187 (2016).

[b64] Serkov A. A., Kuzmin P. G. & Shafeev G. A. Laser-induced agglomeration of gold and silver nanoparticles dispersed in liquid. Chem. Phys. Lett. 647, 68–72 (2016).

[b65] Mafuné F., Kohno J.-y., Takeda Y. & Kondow T. Nanoscale Soldering of Metal Nanoparticles for Construction of Higher-Order Structures. Journal of the American Chemical Society 125, 1686–1687 (2003). 12580579 10.1021/ja021250d

[b66] Link S., Burda C., Mohamed M. B., Nikoobakht B. & El-Sayed M. A. Femtosecond transient-absorption dynamics of colloidal gold nanorods: shape independence of the electron-phonon relaxation time. Phys Rev B 61, 6086 (2000).

[b67] Elsayed-Ali H. E., Norris T. B., Pessot M. A. & Mourou G. A. Time-resolved observation of electron-phonon relaxation in copper. Phys. Rev. Lett. 58, 1212 (1987). 10034371 10.1103/PhysRevLett.58.1212

[b68] Medvedev N., Li Z. & Ziaja B. Thermal and nonthermal melting of silicon under femtosecond X-ray irradiation. Phys. Rev. B 91, 054113 (2015).

[b69] Zhukov V. P., Tyuterev V. & Chulkov E. V. Electron–phonon relaxation and excited electron distribution in zinc oxide and anatase. J. Phys.: Condens. Matter 24, 405802 (2012). 22967967 10.1088/0953-8984/24/40/405802

[b70] Blandin P. . Femtosecond laser fragmentation from water-dispersed microcolloids: toward fast controllable growth of ultrapure Si-based nanomaterials for biological applications. Journal of Materials Chemistry B 1, 2489–2495 (2013). 10.1039/c3tb20285b 32261049

